# Chronic Gastric Ulcer Healing Actions of the Aqueous Extracts of Staple Plant Foods of the North-West, Adamawa, and West Regions of Cameroon

**DOI:** 10.1155/2023/2657278

**Published:** 2023-01-09

**Authors:** Hauberline Domegne Mikwangock, Alfred Ngenge Tamfu, André Perfusion Amang, Gael Tchokomeni Siwe, Christophe Mezui, Selcuk Kucukaydin, Enonchong George Enow-Orock, Paul Vernyuy Tan

**Affiliations:** ^1^Department of Animal Biology & Physiology, Faculty of Science, University of Yaounde I, Yaounde, P.O. Box 812, Cameroon; ^2^Department of Chemical Engineering, School of Chemical Engineering and Mineral Industries, University of Ngaoundere, 454 Ngaoundere, Cameroon; ^3^Department of Biological Sciences, Faculty of Science, University of Maroua, Maroua, P.O. Box 814, Cameroon; ^4^Department of Biological Sciences, Higher Teachers' Training College, University of Yaoundé I, P.O. Box 047, Yaoundé, Cameroon; ^5^Department of Medical Services and Techniques, Koycegiz Vocational School of Health Services, Mugla Sitki Kocman University, Mugla 48800, Turkey; ^6^Department of Biomedical Sciences, Faculty of Health Sciences, University of Buea, Buea, P.O. Box 63, Cameroon

## Abstract

**Aim:**

This study is aimed at establishing phenolic compound profile and assessing the possible antiulcer activities of aqueous extracts of some staple plant foods from the West and North-West regions of Cameroon against chronic gastric ulcer models in rats.

**Materials and Methods:**

Phenolic constituents of extracts were evaluated using HPLC-DAD. Aqueous extracts of *Corchorus olitorius*, *Solanum nigrum*, *Vigna unguiculata*, *Triumfetta pentandra*, “nkui” spices, and “yellow soup” spices were tested at two doses (200 and 400 mg/kg). After treatments, animals were sacrificed, healing percentage and antioxidant status (catalase, superoxide dismutase, and glutathione peroxidase) were evaluated, and histological examination of gastric mucosa was realized.

**Results:**

HPLC-DAD revealed that p-hydroxybenzoic and protocatechuic acids were the phenolic compound present in all extracts. Oral administration of extracts (200 and 400 mg/kg) significantly reduced ulcer surface value and significantly increased mucus production compared to the control groups (*p* < 0.05). Histological study supported the observed healing activity of different extracts characterized by a reduced inflammatory response. Moreover, administration of aqueous extracts increased the activity of antioxidant enzymes.

**Conclusion:**

This study revealed that aqueous extracts of *Solanum nigrum*, *Corchorus olitorius*, *Vigna unguiculata*, *Triumfetta pentandra*, “yellow soup” spices, and “nkui” spices possess healing antiulcer effects against models of gastric ulcers. The antiulcer mechanisms involved may include increase of gastric mucus production and improvement of the antioxidant activity of gastric tissue. These activities may be due to the phenolic compounds identified in the extracts, especially p-hydroxybenzoic and protocatechuic acids present in all extracts and with known antioxidant, cytoprotective, and healing properties. However, all the diets may promote the healing process of chronic ulcers caused by excessive alcohol consumption/stress.

## 1. Introduction

A peptic ulcer is a sore on the inner lining of the stomach or duodenum [1]. Peptic ulcer is a chronical development disease, characterized by an imbalance between the factors that damage the mucosa (*Helicobacter pylori*, acid, pepsin, nonsteroidal anti-inflammatory drugs (NSAIDs), stress, and alcohol) and those for its protection (gastric mucus, bicarbonate, and antioxidants compounds) [2]. Peptic ulcer is a major public health problem, and most of its complications have been the major causes of morbidity and mortality [3]. A number of drugs may be used in treatment of gastric ulcer; proton pump inhibitors (PPI), histamine H_2_ receptor antagonists, and mucosal protective agents can thus all be used as protective drugs against initiation of gastric ulcer in predisposed groups as well as prevention of remittent attacks. A standard therapy consisting of a PPI and two antibiotics (clarithromycin and amoxicillin/metronidazole) is widely used as the first-line regimen for treatment of *Helicobacter pylori* infection [4]. A number of drugs used as antiulcer medications show side effects such as diarrhea, nausea, vomiting, and anemia, and *Helicobacter pylori* strains increasingly show resistance to the antibiotics of choice [5]. The plant kingdom is endowed with alternative sources of therapeutic substances in the form of secondary metabolites (alkaloids, triperpenes, tannins, phenolics, etc.) and minerals with useful therapeutic activities. The presence of these therapeutic substances in significant amounts in the plants that are consumed as staple diets implies that their beneficial curative and/or prophylactic effects can be obtained through regular consumption of specific diets. Thus, while certain foods may have ingredients that fight against the bacteria *Helicobacter pylori*, a main cause of ulcers, some foods like coffee, chocolate, spicy foods, alcohol, and acidic foods such as citrus and tomatoes, may make acid reflux worse. Due to its geographical location, Cameroon is distinguished by a contrasting relief of alternate high and low lands and offers an entire range of intertropical climates. This climatological diversity and the associated sociological specificities have created five distinct agroecological zones from north to south, namely, the Sahelian zone, the low-lying savannah zone, the wet savannah zone, the forest zone, and the mangrove zone. The distinct climate-sociological specificities of these zones have in turn created culinary specificities based on the food crops that are cultivated and regularly consumed in each zone. The literature reveals that, among the useful secondary metabolites present in the plant foods, phenolic compounds possess several health benefits; they are widespread in food plant seeds, fruits, and various edible vegetables and useful for human health through their beneficial antioxidant activities among others [6–10]. The Adamawa, West, and North-West administrative regions of Cameroon are located within the low-lying savannah and wet savannah agroecological zones, whose principal staple diets include dishes prepared using *Solanum nigrum*, *Corchorus olitorius*, *Vigna unguiculata*, and *Triumfetta pentandra*, as well as 11 spices used in the preparation of “nkui” (*Dorstenia psilurus*, *Zanthoxylum zanthoxyloides*, *Scorodophloeus zenkeri*, *Zanthoxylum leprieurii*, *Xylopia aethiopica*, *Xylopia parviflora*, *Mondia whitei*, *Solanum gilo*, *Pentadiplandra brazzeana*, *Tetrapleura tetraptera*, and *Capsicum frutescens*) and 14 spices used in the preparation of “yellow soup” (*Tetrapleura tetraptera*, *Dichrostachys glomerata*, *Afrostyrax lepidophyllus*, *Piper umbellatum*, *Monodora myristica*, *Scorodophloeus zenkeri*, *Aframomum daniellii*, *Xylopia aethiopica*, *Piper guineense*, *Piper capense*, *Echinops giganteus*, *Hua gabonii*, *Capsicum frutescens*, and *Scleria striatinux*) [11, 12]. Previous work showed that when these plants were added to whole diets and fed to experimental rats, the diets had both gastroprotective and curative effects against various models of gastric ulcers in rats [13]. Elsewhere, other workers [14–16] have shown that such effects were attributed to the natural phenolic compounds found in the plant extracts. In the present work, we analysed the phenolic compound content of four plants (*Solanum nigrum*, *Corchorus olitorius*, *Vigna unguiculata*, and *Triumfetta pentandra*) and two spice complexes (“nkui” and “yellow soup”) consumed as staple diets in the Adamawa, West, and North-West regions of Cameroon and tested the healing actions of the aqueous extracts on chronic gastric ulcers induced in experimental rats.

## 2. Material and Methods

### 2.1. Plant Harvesting

The fresh plant parts were harvested between June and September 2019 at Ngaoundere (7°20′17.336^″^N, 13°34′0.588^″^E), Bafoussam (5°28′0.001^″^N, 10°25′0.001^″^E), and Bamenda (5°57′34.92^″^N, 10°08′45.49^″^E) and authenticated by Mr. Ngansop Eric (botanist) at the National Herbarium of Cameroon in comparison with specimens deposited under the voucher numbers indicated in Table 1. They were air-dried in the shade and ground using a mechanical grinder to a fine powder.

### 2.2. Preparation of Extracts

300 grams of powder from *Solanum nigrum*, *Corchorus olitorius*, *Vigna unguiculata*, and *Triumfetta pentandra* samples was each mixed with one liter of hexane, and the mixtures were macerated during 72 hours and filtered through Whatman filter paper No. 4. The filtrate was left to stand at room temperature during 10 hours. These residues were each mixed with one liter of hot water (77°C) for 10 minutes, and the mixture was macerated for 24 hours and then filtered using Whatman paper No. 4. The filtrate obtained was evaporated at 40°C for 48 hours using a ventilation air oven (Jencons-PLS, UK), and the aqueous extracts obtained were stored at room temperature for further use. The constitutive elements (11 and 14, respectively) of the “nkui” and “yellow soup” spice mixtures were compounded following the quantities (per 100 g of mixture) indicated in Table 1, and the aqueous extract of each spice mixture was prepared.

### 2.3. Identification and Quantification of Phenolic Compounds in the Aqueous Extract

The phenolic compounds in plant extracts were detected and quantified using reversed-phase high-performance liquid chromatography (RP-HPLC) coupled with diode array detector (DAD) as described by Cayan et al. [52] and Tamfu et al. [53]. Each aqueous extract was dissolved in a water/methanol mixture (80 : 20) and then filtered on sterile 0.20 *μ*m millipore, and an Inertsil ODS-3 reverse phase C18 column was used for the separation employing a 1.0 mL/min solvent flow rate and 20 *μ*L injection volume. Two mobile phases (A: 0.5% acetic acid in H_2_O and B: 0.5% acetic acid in CH_3_OH were used. A gradient elution was applied as follows: 0-10% B (0-0.01 min), 10-20% B (0.01-5 min), 20-30% B (5-15 min), 30-50% B (15-25 min), 50-65% B (25-30 min), 65-75% B (30-40 min), 75-90% B (40-50 min), and 90-10% B (50-55 min).

A photodiode array detector set at 280 nm wavelength was employed in the detection, and the UV data together with retention times were compared with authentic standards. Each analysis was performed three times. A calibration plot established through the elution of known concentrations (0.0, 0.00782, 0.01563, 0.03125, 0.0625, 0.125, 0.25, 0.5, and 1.0 ppm) of authentic compounds was used in the identification and quantification of the constituent phenolic compounds as described by Cayan et al. [52] and Tamfu et al. [53].

### 2.4. Evaluation of Curative Effect of Extracts on Gastric Ulcers

#### 2.4.1. Induction of Chronic “Unhealed Gastric Ulcers”

Ulcers were induced according to Pillai and Santhakumari's [54] method, with slight modifications as described by Wang et al. [55]. Eighty rats were selected and divided into 16 groups of 5 rats each. These rats were fasted for 24 hours and gastric ulcers were induced except for the normal group. Animals were anesthetized, laparotomy was performed, and 0.05 mL of 30% acetic acid was injected through the serosal surface of the stomachs, and the surface was cleaned with saline (NaCl) solution 0.9%. The abdominal incision was stitched and disinfectant (betadine) was applied daily to avoid infection. On the fourth day after ulcer induction, a group (control 1 or transversal control) was sacrificed under anaesthesia, and their stomachs were opened, and then, ulcer surface and mucus production were measured. One part of the stomach was stored in 10% formalin for histological studies, and the other part was conserved for measurement of antioxidant parameters. The remaining groups were treated for 14 days, from day five after ulcer induction, as follows:

- Group 1: normal group, without ulcer induction, received distilled water (1 mL/200 g)
- Group 2: control 2 (longitudinal control) ulcerated rats which received distilled water (1 mL/200 g)
- Group 3: control 3 (positive control) ulcerated rats that received sucralfate at a dose of 50 mg/kg

Groups 4 and 5 were treated with *Corchorus olitorius*; groups 6 and 7 were treated with *Triumfetta pentandra*; groups 8 and 9 were treated with the *Solanum nigrum*; groups 10 and 11 were treated with *Vigna unguiculata*; groups 12 and 13 were treated with “nkui” soup spices; groups 14 and 15 were treated with “yellow soup” spices. The dose of 200 mg/kg was used for groups 4, 6, 8, 10, and 12. The dose of 400 mg/kg was used for groups 5, 7, 9, 11, and 13.

Subcutaneous injections of indomethacin (1 mg/kg) were concomitantly administered to these groups (2-15) once daily for 14 days. On the 15^th^ day, animals were fasted for 24 hours, sacrificed, and treated following the same steps as described above.

#### 2.4.2. Ethanol-/Stress-Induced Chronic Gastric Ulcers

Ulcers were induced according to the methods described by Sun-Hye et al. [56] and Arai et al. [57]. After 48 h fasting, ulcers were induced (except normal group) by oral administration of ethanol (1 mL/200 g), and one hour after, stress was induced by placing the animals in individual restraint tubes (to restrict movements) and introducing them into cold water (20 ± 1°C), for three hours. For the next three days, the gastric ulcer induction was repeated, but the ethanol administered was 40% and the animals were immersed in water rather for one hour and thirty minutes. After 4 days, two groups (controls 1 and 2) were sacrificed and treated as described previously. The remaining animals were treated for 10 days as follows:

- Group 1: normal group, without ulcer induction, received distilled water (1 mL/200 g)
- Group 2: control 3 (longitudinal control) which received distilled water (1 mL/200 g)
- Group 3: control 4 (positive control) received sucralfate at a dose of 50 mg/kg

Groups 4 and 5 were treated with the *Corchorus olitorius*; groups 6 and 7 were treated with *Triumfetta pentandra*; groups 8 and 9 were treated with *Solanum nigrum*; groups 10 and 11 were treated with *Vigna unguiculata*; groups 12 and 13 were treated with the “nkui” spices; groups 14 and 15 were treated with the “yellow soup” spices. The dose of 200 mg/kg was used for groups 4, 6, 8, 10, and 12. The dose of 400 mg/kg was used for groups 5, 7, 9, 11, and 13.

On the 11^th^ day, animals were fasted for 24 hours, sacrificed, and treated following the same steps as described above.

### 2.5. Histopathological Examination

The stomach fragments stored in formaldehyde (10%) were dehydrated using upgraded ethanol series and embedded in paraffin blocks as described by Baponwa et al. [58]. The tissue was sectioned at a thickness of 5 𝜇m, passed through xylene, rehydrated into a series of degraded ethanol, and stained with haematoxylin and eosin (H&E). The stained sections were observed in the microscope, microphotographs were recorded, and scale bars were inserted in the micrographs.

### 2.6. *In Vivo* Antioxidant Activity and Nitric Oxide (NO) Assay

Briefly, 1 g of the stomach of each animal was crushed and homogenized in tris-HCl buffer (50 mM) to obtain a 20% (*w*/*v*) solution. After homogenate centrifugation at 5000 rpm for 10 min, supernatants were collected and stored at -20°C awaiting dosage of some oxidative stress markers. These markers were measured according to well-established protocols. Malondialdehyde (MDA) concentration was measured as described by Wilbur et al. [59]. Superoxide dismutase (SOD) and catalase (CAT) activities were measured according to protocols of Misra and Fridovichl [60] and Sinha [61], respectively. Reduced glutathione (GSH) levels were measured as per Ellman's [62] method. The nitrite content was quantified by measuring nitrite concentration using the Griess reagent assay as described by Fermor et al. [63].

### 2.7. Statistical Analysis

Statistical analyses were performed using GraphPad® software, version 8.0.1. One-way analysis of variance (ANOVA) followed by Tukey's multiple comparison test was used, and results were considered as significant for *p* values less than 0.05. Values in tables are given as arithmetic means ± standard error on the mean (S.E.M.).

## 3. Results

### 3.1. Phenolic Compounds of the Aqueous Extracts

Out of the twenty-six standard phenolic compounds used in the analyses, fourteen phenolic compounds were detected in aqueous extracts of *Corchorus olitorius*, *Solanum nigrum*, *Vigna unguiculata*, *Triumfetta pentandra*, “nkui” spices, and “yellow soup” spices. The number of phenolic compounds detected differed from one extract to another such as presented in Table 2. In the aqueous extract of *Corchorus olitorius*, eight phenolic compounds were detected, which represents with “nkui” spices, the richest extracts. *Solanum nigrum* and *Triumfetta pentandra* contained seven phenolic compounds each and finally the extracts of “yellow soup” spices and *Vigna unguiculata*had, respectively, six and four phenolic compounds. Among the phenolic compounds identified in *Vigna unguiculata*, syringic acid (8.96 ± 0.27 *μ*g/g) was the most abundant. However, protocatechuic acids were found to be the major phenolic compounds in *Corchorus olitorius* (12.30 ± 0.28 *μ*g/g), *Triumfetta pentandra* (25.36 ± 0.43 *μ*g/g), and *Solanum nigrum* (25.36 ± 0.43 *μ*g/g), and P-hydroxybenzoic acids were the common compounds present in all extracts. *Solanum nigrum* and *Triumfetta pentandra* also contained gallic acid in high quantities (12.55 ± 0.39 *μ*g/g and 12.40 ± 0.35 *μ*g/g, respectively); besides protocatechuic acid and gallic acid, ferulic acid was also present in *Solanum nigrum* in high quantity (15.36 ± 0.48 *μ*g/g). A phenolic compound most present in “yellow soup” spices was pyrocatechol (8.97 ± 0.33 *μ*g/g).

### 3.2. Effect of Extracts on “Unhealed Ulcers”

**Figure 1:**
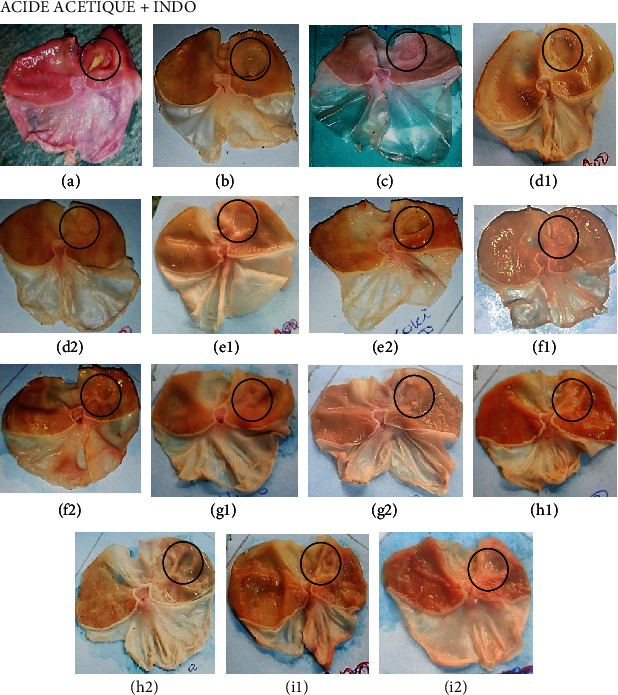
Macroscopic appearance of gastric mucosa in “unhealed ulcers.” (a) Control 1, (b) control 2 (negative control), (c) positive control, and (d–i) the stomachs of rats which received aqueous extract of *Corchorus olitorius*, *Solanum nigrum*, *Vigna unguiculata*, *Triumfetta pentandra*, “nkui” spices, and “yellow soup” spices at doses of 200 (1) and 400 mg/kg (2), respectively. (Black circles indicate ulcerous craters.)

Table 3 presents the effects of extracts on ulcer parameters in animals subjected to “unhealed ulcers.” Figure 1 shows the macroscopic appearance of the stomachs after induction and treatment of ulcers. The stomach of rats, sacrificed 4 days after induction (control 1), had a deep crater (Figure 1(a)). The longitudinal control group (control 2) experienced a crater reduction (Figure 1(b)) compared to the transverse control (control 1), corresponding to a self-healing of 26.32%. Animals treated with sucralfate (50 mg/kg) (Figure 1(c)) also experienced a significant decrease (*p* < 0.001) in ulceration area. Extracts of “nkui” spices (doses of 200 mg/kg) (Figure 1(g1)) and *Corchorus olitorius* (400 mg/kg) (Figure 1(d2)) induced a significant decrease (*p* < 0.05 and *p* < 0.001, respectively) of ulcerated area with healing percentages of 47.34% and 60.38%, respectively.

### 3.3. Effect of Aqueous Extracts on Ethanol-/Stress-Induced Gastric Ulcers

**Figure 2:**
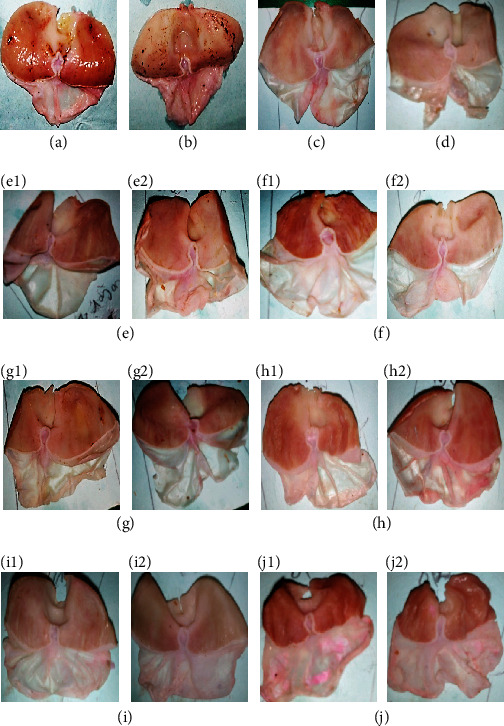
Macroscopic appearance of gastric mucosa in ethanol-/stress-induced model. (a) Control 1, (b) control 2, (c) control 3, (d) positive control, and (e–j) stomachs of rats which received aqueous extract of *Corchorus olitorius*, *Solanum nigrum*, *Vigna unguiculata*, *Triumfetta pentandra*, “nkui” spices, and “yellow soup” spices at 200 (1) and 400 mg/kg (2), respectively.

Figure 2 shows the stomachs of rats after ethanol-/stress-induced ulcers. Control 1 group animals had ulcers confirmed by the presence of hemorrhagic streaks. Figures 2(a) and 2(b) represent the stomachs of rats sacrificed 4 days after ulcer induction, with visible ulcers. In Figure 2(d), ulcerated surface was lower compared to Figure 2(c). There was a significant reduction of ulcer development in groups receiving the aqueous extracts at doses of 200 and 400 mg/kg. This reduction was significative (compared to control 3) in all extract-treated groups as indicated by reduction of ulcerated surface (Table 4). In groups treated with *Triumfetta pentandra* (200 mg/kg), *Solanum nigrum*, and “nkui” spices (400 mg/kg), healing was associated with an increase of mucus secretion (*p* < 0.01) compared to control 3.

### 3.4. Effect of Aqueous Extracts on Oxidative Stress Parameters and NO Level in Gastric Tissues

#### 3.4.1. Effects of Aqueous Extracts on some Parameters of Oxidative Stress and NO Level in Gastric Tissues in “Unhealed Ulcer” Model

Table 5 presents the effects of aqueous extracts on some parameters of oxidative stress in “unhealed ulcer” model. The concentration of MDA increased significantly in animals in the aqueous “nkui” spice extract group (200 and 400 mg/kg) compared to the longitudinal control group. Aqueous extracts of *Triumfetta pentandra* (400 mg/kg) and “nkui” spices (200 and 400 mg/kg) caused a significant increase (*p* < 0.05 and *p* < 0.01) of GSH compared to the longitudinal control. In the positive control group, catalase and SOD oncentrations increased significantly compared to the longitudinal control, and in those treated with the aqueous extract of *Vigna unguiculata* (200 mg/kg) and *Corchorus olitorius* (400 mg/kg), SOD increased significantly compared to the longitudinal control.

#### 3.4.2. Effect of Aqueous Extracts on some Parameters of Oxidative Stress and NO Level in Gastric Tissues in Ethanol-/Stress-Induced Ulcers

Table 6 presents the effects of extracts on some parameters of oxidative stress in animals subjected to ulcer induced with ethanol/stress. The concentration of MDA increased significantly in animals in the sucralfate group (50 mg/kg). Aqueous extract of *Vigna unguiculata* (200 mg/kg); “yellow soup” spices (200 mg/kg), *Triumfetta pentandra* (400 mg/kg) and “nkui” spices (200 and 400 mg/kg), significantly increased GSH levels compared to the longitudinal control group. Aqueous extracts of “yellow soup” spices, “nkui” spices and *T. pentadra* at a dose of 400 mg/kg caused a significant increase (*p* < 0.001) in catalase compared to the longitudinal control. In the group of rats treated with the aqueous extract of *Solanum nigrum* (400 mg/kg), it was observed a significant increase in SOD concentration compared to the longitudinal control, and treatment with *Triumfetta pentandra* (400 mg/kg) induced a significant increase in nitrite concentration compared to the longitudinal control.

### 3.5. Effect of Aqueous Extracts on Stomach Histology

**Figure 3:**
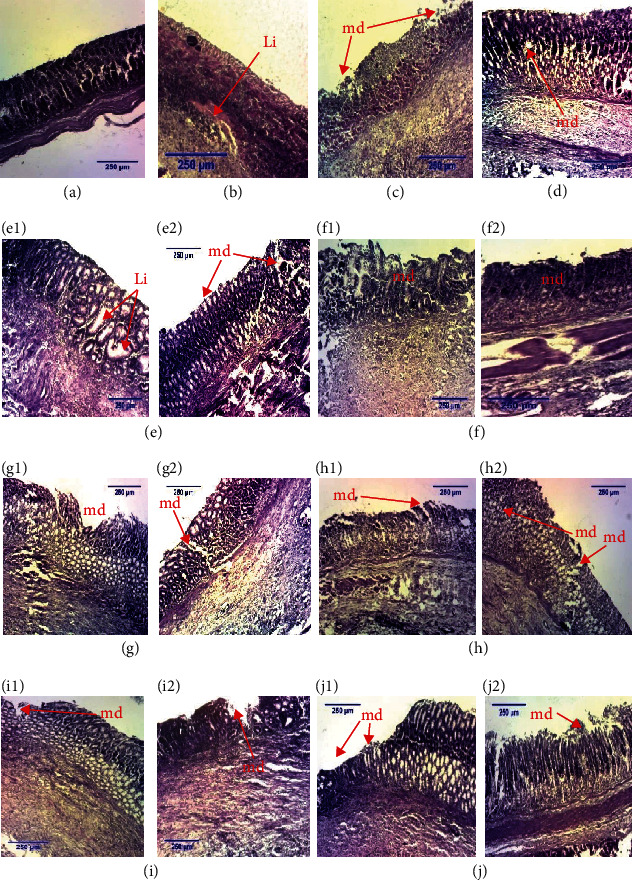
Histological sections of stomachs in acetic acid-/indomethacin-induced model (H&E; ×40). (a) Normal stomach, (b) control 1, (c) control 2 (negative control), (d) positive control, and (e–j) stomachs of rats which received aqueous extract of *Corchorus olitorius*, *Solanum nigrum*, *Vigna unguiculata*, *Triumfetta pentandra*, “nkui” spices, and “yellow soup” spices at dose 200 (1) and 400 mg/kg (2), respectively. md = mucosa destroyed; Li = leukocyte infiltration; mr = tissue regeneration.

Microscopic aspects of rats' stomachs in “unhealed ulcer” model are shown in Figure 3. Figure 3(a) shows section of a normal rat stomach: gastric wall appears normal with all layers, four regions of the stomach, mucosa, submucosa, muscularis, and serosa layers. Figure 3(b) shows severe mucosa destruction of the stomach. Stomach of control 3 (Figure 3(c)) showed partial mucosal regeneration and mucosal destruction (md). In Figure 3(d), the stomach of the sucralfate (50 mg/kg) group showed epithelial proliferation (mr). In Figures 3(e)–3(j), rat stomach sections show a partial mucosal destruction (md) and epithelial reconstitution (mr), but there was persistent leukocyte infiltration in the mucosa (Figures 3(g1), 3(i2), and 3(j2)).

**Figure 4:**
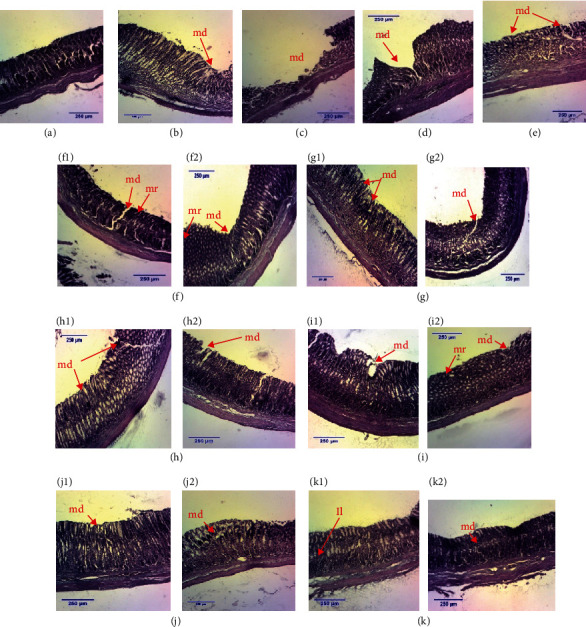
Histological sections of stomachs in ethanol-/stress-induced model (H&E; ×40). (a) Normal stomach, (b) control 1, (c) control 2, (d) control 3 (negative control), (e) positive control, and (f–k) stomachs of rats which received aqueous extract of *Corchorus olitorius*, *Solanum nigrum*, *Vigna unguiculata*, *Triumfetta pentandra*, “nkui” spices, and “yellow soup” spices at dose 200 (1) and 400 mg/kg (2), respectively. md = mucosa destroyed; mr = tissue regeneration; Li = leukocyte infiltration.

Histological sections of stomachs in ethanol-/stress-induced gastric ulcer model are depicted in Figure 4. Figure 4(a) shows the stomach of a nonulcerous animal, showing a healthy, regular, and well-arranged tissue. Controls 1 and 2 (Figures 4(b) and 4(c)) show advanced epithelial destruction with loss of substance of gastric tissue. In Figure 4(d) (control 3), epithelial destruction is still perceptible but with onset of regeneration at epithelial as well as deeper layers. Figure 4(e) represents the stomach of sucralfate-treated rat (50 mg/kg) showing ulcerous surfaces coupled with progressive epithelial layer regeneration. There was also an extract-dependent and dose-dependent reconstruction of different stomach layers in extract-treated groups (Figures 4(d)–4(k)) (200 and 400 mg/kg, respectively).

## 4. Discussion

To investigate the curative effects of aqueous extracts of *Corchorus olitorius*, *Solanum nigrum*, *Vigna unguiculata*, *Triumfetta pentandra*, “nkui” spices, and “yellow soup” spices on gastric ulcers, experimental ulcers were induced by acetic acid/indomethacin (“unhealed ulcers”) and ethanol/stress models. In the acetic acid/indomethacin gastric ulcer induction, acetic acid injected through the serosal surface of stomach diffuses through the disrupted gastric mucosa to induce deep-seated mucosal necrosis and gastric ulcers. Indomethacin causes gastrointestinal injury by blocking prostaglandin synthesis through inhibition of the cyclooxygenase enzymes (COX-1 and COX-2) [64]. In this study, *Corchorus olitorius* (400 mg: 60.28% healing), *Solanum nigrum* (400 mg: 32.13% healing), and “nkui” spices (200 mg: 47.34% healing) were the most active against unhealed ulcers. Even though mucus production remained unchanged compared with normal rats, *Vigna unguiculata*, *Triumfetta pentandra*, and “yellow soup” spices had little or no healing effect up to 400 mg/kg dose. *Solanum nigrum* and *Vigna unguiculata* were most efficient in raising mucus production to 182.5-230.6 mg compared with both the control and normal rats. In previous work, *Triumfetta pentandra* and *Vigna unguiculata* had healing rates of 41.9% and 24.3%, respectively, against “unhealed ulcers” when added to whole diets [13]. These results reveal that *Triumfetta pentandra*, *Vigna unguiculata*, and “yellow soup” spices are more effective in healing of NSAID-induced gastric ulcers when taken in the whole diet compared with the aqueous extracts. A review of the literature (Table 1) shows that all the ingredients involved in the preparation of “nkui” and “yellow soup” spices are endowed with various therapeutic properties including cytoprotective, antiulcerogenic, antioxidant, radical scavenging, anti-inflammatory, and mucogenic activities. In addition, the “yellow soup” spice complex includes *Capsicum frutescens*, *Piper capense*, *and Scleria striatinux* whose anti-*Helicobacter pylori* activities have been shown [32, 43, 46]. The gastroprotective and healing actions of *Corchorus olitorius* and *Solanum nigrum* [47, 49] and the antioxidant activities of *Triumfetta pentandra*, and *Vigna unguiculata* [48, 50, 51] have also been reported. Siwe et al. [65] and Mezui et al. [66] have also reported similar ulcer-healing effects with *Eremomastax speciosa* and *Corchorus olitorius* extracts against “unhealed” gastric ulcers. *Xylopia aethiopica* and *T. tetraptera* fruits in the “nkui” and “yellow soup” spices have shown antiulcer actions against indomethacin [22, 29], and *Dorstenia psilurus* (“nkui” spices) inhibited the formation of gastric lesions in rats [17]. These reports are in support of the ulcer-healing activities observed in the present study.

The ethanol/stress model reproduces the type of ulcer developed by humans following stress, associated with high alcohol consumption. Excessive alcohol consumption is one of the leading causes of acute damage to the gastric mucosa in humans [67, 68]. The mechanism by which ethanol induces gastric ulcers is varied; ethanol causes disruption of the integrity of the gastric mucous barrier by exfoliation of cells, thereby increasing the permeability of mucous membranes to ulcerative agents and in some cases causing bleeding [69, 70]. Stress may promote peptic ulcer through increased acid load, effects of hypothalamic-pituitary-adrenal axis activation of unhealing, altered blood flow, or cytokine-mediated impairment of mucosal defenses [71]. In the present study, administration of ethanol solution by intragastric gavage (four days) produced damage in the gastric mucosa of rats. This damage is well developed when, an hour after the administration of ethanol, rats are subjected to cold water stress with restriction of movements. All the extracts significantly promoted the healing of ethanol-/stress-induced ulcers, and healing effects were more significant in groups treated with *T. pentandra* and *Solanum nigrum* (400 mg/kg), with healing percentages of 98.19 and 98.66%, respectively. The results are in consonance with those achieved with the ethanolic root bark extract of *Zanthoxylum zanthoxyloides* (“nkui” spice) and aqueous seed extract of *Monodora myristica* (“yellow soup” spice) [19, 39]. Iberê et al. [37] also showed similar results with the hydroethanolic extract of *Piper umbellatum* (“yellow soup” spice) on acidified ethanol-induced ulcer. Al Batran et al. [47] also showed healing action of ethanolic extract of *Corchorus olitorius* on ethanol-induced gastric ulcers in adult Sprague Dawley rats. Moreover, oral administration of aqueous extracts of red chilli pepper (“nkui” and “yellow soup” spices) at a dose of 600 mg/kg caused high decrease in the length of gastric ulcers induced by aspirin in rats [31]. In rats treated with *Triumfetta pentandra* (200 mg/kg), *Solanum nigrum*, and “nkui” spices (400 mg/kg), healing was associated with a significant increase of mucus secretion [22, 47]. Results of this study showed that healing potentials for the same extracts at the same doses were higher in ethanol/stress ulcer model compared to “unhealed ulcer” model. This may be explained by the well-known ability of indomethacin to aggravate and delay the healing process of chronic gastric ulcers.

In the “unhealed ulcer” model, mucosal necrosis involves disruption of the integrity of the gastric mucous barrier, and cytokine-mediated impairment of mucosal defenses in ethanol/stress model enhances reactive oxygen species release. Reactive oxygen species play an important role in occurrence and persistence of gastric ulcer [72–74]; they may oxidize cellular proteins and lipids and cause general damage and initiate cell death by apoptosis and necrosis and enhance the development of gastric ulcers. Inhibition of reactive oxygen oxidation activities, lipid peroxidation, and increased antioxidant production can promote gastric ulcer healing. Ulcer healing observed in rats treated with extracts in this study was associated with significant increase of endogenous antioxidants. Previous studies have shown that antioxidants may be connected to antiulcer activity [75]. In “unhealed ulcer” model, “nkui” spices, and *Corchorus olitorius* (200 and 400 mg/kg, respectively) increased GSH concentration. *Corchorus olitorius* (400 mg/kg), compared to longitudinal control, increased SOD concentration. In the ethanol/stress model, “yellow soup” spices, “nkui” spices, and *T. pentandra* extracts at the dose of 400 mg/kg increased catalase concentration compared to the longitudinal controls. Rats treated with the aqueous extract of *Solanum nigrum* (400 mg/kg) showed a significant increase in SOD concentration. These results are in line with those obtained by Iberê et al. [37] who showed that the hydroethanolic extract of *Piper umbellatum* (30, 100, and 300 mg/kg) significantly increased SOD, GSH, and CAT activity. Several studies indicate that SOD appears to be useful in improving the rate of wound healing [76–78]. Therefore, healing actions observed in the treated animals could be due to the ability of the extracts to strengthen the antioxidant defenses of the stomach, promoted by bioactive molecules in the extracts. Ndhlala et al. [79] attributed such antioxidant bioactivity to the presence of phenolics in the plants. Many studies support the contribution of polyphenols in the prevention of peptic ulcer. Polyphenols display a number of pharmacological properties in the gastrointestinal tract area, acting as antisecretory, cytoprotective, and antioxidant agents [80]. Some potent antiulcer plants, such as *Oroxylum indicum*, *Zingiber officinale*, *Olea europaea* L., *Foeniculum vulgare*, *Alchornea glandulosa*, and *Tephrosia purpurea*, contain phenolic compounds, namely, baicalein, cinnamic acid, oleuropein, rutin, quercetin, and tephrosin, respectively, as active constituents [80]. Analysis of the aqueous extracts of *Corchorus olitorius*, *Solanum nigrum*, *Vigna unguiculata*, *Triumfetta pentandra*, “nkui” spices, and “yellow soup” spices revealed that these extracts possess diverse bioactive phenolic compounds. GC-MS analysis showed the presence of 17 different phytochemical compounds in the hydroethanolic extract of *Piper umbellatum*, and HPLC analysis confirmed the presence of quercetin, protocatechuic acid, and ferulic acids in the hydroethanolic extract of *Piper umbellatum* [38, 81]. Despite that the number of phenolic compounds identified in this study differs from one extract to another, p-hydroxybenzoic and protocatechuic acids were the compounds present in all extracts, and two phenolic acids occurring naturally in the plant kingdom possess pharmacological activities. Merkl et al. [82] and Kim et al. [83] reported that p-hydroxybenzoic acid and protocatechuic acid possess good antioxidant effects. The curative effect of these extracts could therefore be due, in part, to their ability to enhance the stomach's antioxidant defenses. Apart from these activities, p-hydroxybenzoic acid (4-hydroxybenzoic acid) and protocatechuic acid also exert other important anti-inflammatory effects. Anti-inflammatory activity of p-hydroxybenzoic acid was reported by Luecha et al. [84] and Manuja et al. [85]. Wei et al. [86] and Wang et al. [87] also reported that protocatechuic acid inhibited inflammatory cytokine tumor necrosis factor-*α* (TNF-*α*), interleukin-1 *β* (IL-1*β*), and IL-6 through NF-*κ*B inhibition pathway. These two phenolic acids could therefore play an important role in the healing process of gastric ulcers observed in this study. However, in addition to these compounds, several others have been identified whose biological properties related to the healing of gastric ulcers have been demonstrated: ferulic acid found in *Corchorus olitorius* and catechin found in *Solanum nigrum* both have a strong positive correlation between radical scavenging activities and reducing power [88]. Gallic acid present in “nkui” spices, *Triumfetta* pentandra, and *Solanum nigrum* is known to inhibit lipid peroxidation [89]. Quercetin, present in *Triumfetta pentandra* extract, is a compound with strong antioxidant activity, high ability to scavenge free radicals, and ability to inhibit lipid peroxidation [90, 91]. Caffeic acid, a phenolic compound identified in “nkui” spices, can delay the appearance of oxidation secondary products and can fight more effectively against lipid oxidation [92]. Hence, these plant secondary metabolites detected in these extracts could play a significant role in the gastric ulcer healing process.

Nitric oxide (NO) also plays an important role in the defense of the gastric mucosa [93]. It is secreted by endothelial cells and nerves of the enteric nervous system that contain nitric oxide synthase (NO_S_) [94]. Increased NO in gastric tissue would improve blood circulation in the gastric mucosa and would also promote angiogenesis, which is one of the major keys to the treatment of gastric ulcers [95]. Treatment with *Vigna unguiculata*, *T. pentandra*, “yellow soup” spices extracts (200 mg/kg), and “nkui” spices extracts (400 mg/kg) induced a significant increase of nitrite levels in the ethanol/stress ulcer model. These results suggest that the curative effect of these extracts may also be due to its ability to improve blood circulation and promote angiogenesis through increased NO production.

Treatment of gastric ulcers aims not only to relieve pain but also to generate scar tissue formation, thus preventing the recurrence of the disease that can lead to complications. Scar tissue formation is a phenomenon of regeneration, a process by which tissue and organ damage is repaired [96]. Scar tissue formation process has 4 phases: the inflammatory phase during which the inflammatory reaction causes vasodilation and increases the permeability of blood vessels at the level of the lesion; the proliferative phase which is the massive proliferation of cells, blood vessels, and fibers; the contraction and epithelialization phase, where under the crust all the connective tissue cells migrate to the center of the lesion; and the scar remodeling phase during which the crust falls and the mucosa will regain its different layers. However, healing disorders can cause disharmonious scars leading to the recurrence of the disease. In acetic acid-/indomethacin- and ethanol-/stress-induced ulcers, histopathological examination revealed the presence of craters in control rats treated with distilled water. The histological section of the stomach of the animals subjected to gastric ulceration induced by acetic acid/indomethacin and treated with the aqueous extracts of *Corchorus olitorius* (200 mg/kg), *Solanum nigrum* (200 and 400 mg/kg), *Vigna unguiculata*, *Triumfetta pentandra* (200 and 400 mg/kg), “nkui” spices (200 and 400 mg/kg), and “yellow soup” spices (200 mg/kg) showed reduced ulcer craters and active cell proliferation. Rats treated with aqueous extracts of “nkui” spices and *Corchorus olitorius* (200 and 400 mg/kg, respectively) showed little leukocyte infiltration and reepithelialization of gastric tissue. In animals subjected to ethanol-/stress-induced gastric ulcers, treatment with *Solanum nigrum* aqueous extract, “yellow soup” spices, *Corchorus olitorius*, and “nkui” spices at doses of 200 and 400 mg/kg and *Vigna unguiculata* (200 mg/kg) showed high reduction of number of inflamed sites and very shallow craters. Previous studies also showed that the plant extracts could protect the gastric mucosa and reduce leukocyte infiltration into the gastric wall in rats [47]. These observations reveal that the extracts accelerate the healing of gastric ulcers induced by acetic acid/indomethacin and ethanol/stress not only by reducing the infiltration of neutrophils into the gastric tissue but also by accelerating the self-healing of gastric tissue, which could prevent the recurrence of the disease. These results corroborate with those obtained by Fouad et al. [97] with hyaluronate on indomethacin-induced gastric ulcers, where they observed that reducing neutrophil infiltration into gastric tissue accelerates the healing of gastric ulcers in rats.

## 5. Conclusion

This study revealed that aqueous extracts of *Solanum nigrum*, *Corchorus olitorius*, *Vigna unguiculata*, *Triumfetta pentandra*, “yellow soup” spices, and “nkui” spices possess antiulcer healing effects against “unhealed” and alcohol/stress ulcer models. The mechanisms involved may include increase of gastric mucus production and improvement of the antioxidant activity of gastric tissue. These activities may be due to the phenolic compounds identified in the extracts, especially P-hydroxybenzoic and protocatechuic acids present in all extracts and with known antioxidant, cytoprotective, and healing properties. The results suggest that regular consumption of *Vigna unguiculata* and *Triumfetta pentandra* staple diets may not be advisable for peptic ulcer patients who are on NSAID prescription. However, all the diets may promote the healing process of chronic ulcers caused by excessive alcohol consumption/stress.

## Figures and Tables

**Table 1:** Plants used in the preparation of experimental diets.

Species (families)	Part of plant used (g quantity for 100 g of spice complexes)	Voucher no.	Metabolites and previously reported activities
Spices used in the preparation of “nkui”			
*Dorstenia psilurus* Welw. (Moraceae)	Roots (4)	3488 SRFCam	(i) Cytoprotective effect of the aqueous extract of *Dorstenia psilurus* roots on gastric ulcer in male rats of the Wistar strain [17]
*Zanthoxylum zanthoxyloides* (Lam.) Zepern. &Timler (Rutaceae)	Fruits (15)	21793 SRFCam	(i) Evaluated the chemical composition of the spices used in the preparation of the soup and “nkui”, among them *Fagara leprieurii* [12] (ii) Analysis of essential fruit oils showed a predominance of alpha-pinene (38.2%) [18] (iii) Gastroprotective effect and safety assessment of *Zanthoxylum zanthoxyloides* root bark [19]
*Scorodophloeus zenkeri* Harms (Caesalpiniaceae)	Fruits (4)	52727 SRFCam	(i) Phenolic compounds and radical scavenging potential of twenty Cameroonian spices including *Scorodophloeus zenkeri* [20]
*Zanthoxylum leprieurii* Guill. &Perr. (Rutaceae)	Seeds (3)	66842 HNC	(i) Evaluation of the antioxidant and anti-inflammatory potential of the essential oils of some Zanthoxylum (Rutaceae) of Cameroon [21] (ii) Evaluation of the chemical composition of the spices entering the preparation of nah-poh “yellow soup” and “nkui,” including *Fagara leprieurii* [12]
*Xylopia aethiopica* (Dunal) A. Rich. (Annonaceae)	Fruits (2)	55011 SRFCam	(i) Antiulcerogenic and mucogenic activity of *Xylopia aethiopica* fruit extract in rat [22] (ii) Chemical composition of seed and oil of *Xylopia aethiopica* growth in Nigeria [23] (iii) Antioxidant and anti-inflammatory activities in human gastric epithelial cells of hydroalcoholic extract obtained from *Xylopia aethiopica* [24]
*Xylopia parviflora* (A. Rich.) Benth. (Annonaceae)	Fruits (2)	42349 SRFCam	(i) Antioxidant and anti-inflammatory activities in human gastric epithelial cells of hydroalcoholic extract obtained from *Xylopia aethiopica* [24]
*Mondia whitei* (Hook.f.) Skeels (Apocynaceae)	Roots (30)	34180 SRFCam	(i) Phenolic compounds and radical scavenging potential of twenty Cameroonian spices including *Mondia whitei* [20] (ii) Antioxidant activity of *Mondia whitei* (Hook. f.) Skeels [25]
*Solanum gilo* Raddi (Solanaceae)	Fruits (10)	14602 SRFCam	(i) Alkaloids, flavonoids, glycosides, terpenoids, and phenolic compounds [26–28]
*Pentadiplandra brazzeana* Baill. (Pentadiplandraceae)	Roots (13)	10384 SRFCam	(i) Phenolic compounds and radical scavenging potential of twenty Cameroonian spices including *Pentadiplandra brazzeana* [20]
*Tetrapleura tetraptera* (Schum. &Thonn.) Taub. (Mimosaceae)	Bark (14) and fruits (2)	44812 SRFCam	(i) Gastroprotective activity of fruit ethanolic extract of *Tetrapleura tetraptera* on indomethacin-induced ulcer in rats [29] (ii) Antioxidant and anti-inflammatory activities in human gastric epithelial cells of hydroalcoholic extract obtained from *Tetrapleura tetraptera* [24]
*Capsicum frutescens* L. (Solanaceae)	Fruits (1)	18629 SRFCam	(i) Changes in phytochemical and antioxidant activity of selected pepper cultivars (Capsicum species) as influenced by maturity [30] (ii) Antiulcer activity of aqueous extract on aspirin-induced ulcer [31] (iii) Antiulcer activity of capsaicin on *H. pylori*-infected stomachs [32]
Spices used in the preparation of “yellow soup”			
*Tetrapleura tetraptera* (Schum. &Thonn.) Taub. (Mimosaceae)	Fruits (12)	44812 SRFCam	(i) Gastroprotective activity of fruit ethanolic extract of *Tetrapleura tetraptera* on indomethacin-induced ulcer in rats [29] (ii) Antioxidant and anti-inflammatory activities in human gastric epithelial cells of hydroalcoholic extract obtained from *Tetrapleura tetraptera* [24]
*Dichrostachys glomerata* (Forssk.) Chiov. (Mimosaceae)	Fruits (3)	43974 SRFCam	(i) Phenolics detected among group of compounds include 2-methoxy phenol, 2-methoxyl-4-vinylphenol, and 2,4-bis (1,1-dimethylethyl) phenol [33] (ii) Antioxidant and anti-inflammatory activities of hydroalcoholic extracts of *Dichrostachys glomerata* in gastric epithelial cells [24]
*Afrostyrax lepidophyllus* Mildbr. (Huaceae)	Seeds (3)	61286 HNC	(i) Thioglycosides in seeds, 2,4,5,7-tetrathiaoctane, and other S-containing compounds in the essential oil from seeds [34, 35]
*Piper umbellatum* L. (Piperaceae)	Fruits (2)	56269 SRFCam	(i) Agbor et al. [36] reported the antioxidant capacity of *Piper umbellatum* (ii) Leaf hydroethanolic extract of *P. umbellatum* has gastroprotective and ulcer healing against acidified ethanol (acute) and acetic acid (chronic) gastric ulcer models in rodents [37] (iii) Phytochemical profiles of the hydroethanolic leaf extract and intestinal anti-inflammatory mechanisms on 2,4,6-trinitrobenzene sulfonic acid-induced ulcerative colitis in rats [38]
*Monodora myristica* (Gaertn.) Dunal (Annonaceae)	Almond (3)	1927 SRFCam	(i) *M. myristica*: review: a plant with multiple food, health, and medicinal applications [39] (ii) Antiulcer activity higher against alcohol than aspirin model [40]
*Scorodophloeus zenkeri* Harms (Caesalpiniaceae)	Barks and fruits (10)	44803 SRFCam	(i) Phenolic compounds and radical scavenging potential of twenty Cameroonian spices including *Scorodophloeus zenkeri* [20]
*Aframomum daniellii* (Hook. f.) K. Schum. (Zingiberaceae)	Fruits (8)	60957 HNC	(i) Phenolic compounds and radical scavenging potential of twenty Cameroonian spices including: *Aframomum daniellii* [20]
*Xylopia aethiopica* (Dunal) A. Rich. (Annonaceae)	Fruits (2 g)	45573 SRFCam	(i) Antiulcerogenic and mucogenic activity of *Xylopia aethiopica* fruits extract in rat [22] (ii) Chemical composition of seed and oil of *Xylopia aethiopica* growth in Nigeria [23] (iii) Antioxidant and anti-inflammatory activities in human gastric epithelial cells of hydroalcoholic extract obtained from *Xylopia aethiopica* [24]
*Piper guineense* Schum. &Thonn. (Piperaceae)	Fruits (2)	19991 SRFCam	(i) Antiulcer activity of black pepper against absolute ethanol-induced gastric mucosal damage in mice [41]
*Piper capense* L.f. (Piperaceae)	Fruits (1)	57989 HNC	(i) Piper species: a comprehensive review on their phytochemistry, biological activities, and applications including *Piper capense* [42] (ii) Inhibitory effect of piperine on *Helicobacter pylori* growth and adhesion to gastric adenocarcinoma cells [43]
*Echinops giganteus* A. Rich. (Asteraceae)	Roots (23)	7957 SRFCam	(i) The genus Echinops: phytochemistry and biological activities: a review [44] (ii) Antioxidant activity [45]
*Hua gabonii* Pierre (Huacaceae)	Fruits (3) and bark (27)	66306 HNC	(i) Phenolic compound and radical scavenging potential of twenty Cameroonian spices including *Hua gabonii* (fruits) [20] (ii) Chemical composition of spices used in the preparation of “nah-poh” and “nkui” from Western Cameroon [12]
*Capsicum frutescens* L. (Solanaceae)	Fruits (1)	18629 SRFCam	(i) Changes in phytochemical and antioxidant activity of selected pepper cultivars (Capsicum species) as influenced by maturity [31] (ii) Antiulcer activity of aqueous extract on aspirin-induced ulcer [31] (iii) Antiulcer activity of capsaicin on *H. pylori*-infected stomachs [32]
*Scleria striatinux* De Wild. (Cyperaceae)	Roots (2)	42536 SRFCam	(i) *In vitro* anti-*Helicobacter pylori* activity of extracts of selected medicinal plants from North-West Cameroon [46]. Tchiegang and Mbougueng [12] made a chemical characterization

Other plants tested			
*Corchorus olitorius* L. (Tiliaceae)	Leaves	51779 SRFCam	(i) Ethnonutritional data and physical-chemical characteristics of leafy vegetables consumed in the savannah (Cameroon) [11] (ii) Gastroprotective effects of *Corchorus olitorius* leaf extract against ethanol-induced gastric mucosal hemorrhagic lesions in rats [47]
*Triumfetta pentandra* A. Rich (Malvaceae)	Bark	27523 SRFCam	(i) Antioxidant and antimicrobial properties of a culinary plant in the Western Cameroon region: cases of *Triumfetta cordifolia* [48]
*Solanum nigrum* L. (Solanaceae)	Leaves and stem	31965 SRFCam	(i) Antiulcerogenic and ulcer-healing effects of *Solanum nigrum* (L.) on experimental ulcer models: possible mechanism for the inhibition of acid formation [49] (ii) Ethnonutritional data and physical-chemical characteristics of leafy vegetables consumed in the savannah (Cameroon) [11]
*Vigna unguiculata* (L.) Walp. (Fabaceae)	Seeds	42580 SRFCam	(i) Ethnonutritional data and physical-chemical characteristics of leafy vegetables consumed in the savannah (Cameroon) [11] (ii) Characterization of bioactive phenolics and antioxidant capacity of edible bean extracts of 50 Fabaceae populations grown in Thailand [50] (iii) Wound healing plants in Mali, the Bamako region. An ethnobotanical survey [51]

**Table 2:** Phenolic composition of the aqueous extracts.

No.	Phenolic compounds (*μ*g/g)	Retention time (min)	*Corchorus olitorius*	*Vigna unguiculata*	“nkui” spices	*Triumfetta pentandra*	*Solanum nigrum*	“Yellow soup” spices
1	Gallic acid	5.70	3.43 ± 0.12	—	8.86 ± 0.14	12.40 ± 0.35	12.55 ± 0.39	2.25 ± 0.14
2	Protocatechuic acid	8.75	12.30 ± 0.28	3.21 ± 0.14	3.30 ± 0.25	25.36 ± 0.43	9.44 ± 0.31	3.05 ± 0.12
3	Catechin	10.68	—	—	4.46 ± 0.20	—	22.31 ± 0.42	—
4	Pyrocatechol	11.04	4.50 ± 0.20	—	—	—	—	8.97 ± 0.33
5	Chlorogenic acid	12.35	—	—	3.24 ± 0.33	—	—	
6	P-hydroxybenzoic acid	12.77	3.77 ± 0.31	2.43 ± 0.10	2.28 ± 0.22	5.84 ± 0.32	3.58 ± 0.29	2.98 ± 0.19
7	Caffeic acid	15.09	—	—	2.14 ± 0.11		—	—
8	3-Hydroxybenzoic acid	15.98	—	2.85 ± 0.15	2.20 ± 0.17	6.96 ± 0.28	—	4.73 ± 0.15
9	Syringic acid	16.56	—	8.96 ± 0.27	4.05 ± 0.24	—	7.78 ± 0.26	5.23 ± 0.24
10	Ferulic acid	22.14	3.14 ± 0.17	—	—	—	15.36 ± 0.48	—
11	Ellagic acid	26.11	3.12 ± 0.11	—	—	4.33 ± 0.16	—	—
12	Rosmarinic acid	26.77	5.28 ± 0.21	—	—	7.83 ± 0.27	—	—
13	Quercetin	30.83	—	—	—	4.46 ± 0.22	—	—
14	Luteolin	31.70	3.05 ± 0.16	—	—	—	5.43 ± 0.25	—

Values expressed are means ± S.E.M. of three measurements; —: not detected.

**Table 3:** Effect of aqueous extracts on “unhealed ulcers”.

Groups	Parameters			
	Doses (mg/kg)	Ulcerated surface (mm^2^)	% Healing	Mucus production (mg)
Normal	—	—	—	137.08 ± 4.83
Control 1	—	49.00 ± 1.78	—	138.33 ± 3.60
Control 2	—	36.10 ± 1.72	26.32	205.25 ± 6.62
Control 3 (sucralfate)	50	9.40 ± 0.57^∗∗∗^^,abc^	73.96	143.60 ± 2.10
*Corchorus olitorius*	200	182.05 ± 2.30^abc^	0	107.11 ± 5.81
*Corchorus olitorius*	400	14.30 ± 0.31^∗∗∗^^,abc^	60. 38	172.50 ± 0.15
*Solanum nigrum*	200	51.20 ± 0.31	0	102.09 ± 4.11
*Solanum nigrum*	400	24.50 ± 0.15^abc^	32.13	182.50 ± 0.47
*Vigna unguiculata*	200	88.12 ± 0.63^abc^	0	230.61 ± 3.92^∗∗^
*Vigna unguiculata*	400	67.10 ± 4.11^a^	0	122.5 ± 6.48
*Triumfetta pentandra*	200	83.23 ± 0.94^abc^	0	124.07 ± 4.34
*Triumfetta pentandra*	400	36.11 ± 3.16	0	155.10 ± 5.06
“nkui” spices	200	19.01 ± 3.47^∗^^,abc^	47.34	146.50 ± 1.73
“nkui” spices	400	30.05 ± 2.02^ab^	16.75	134.02 ± 1.58
“Yellow soup” spices	200	53.15 ± 0.94	0	157.20 ± 4.74
“Yellow soup” spices	400	33.50 ± 2.68	7.20	144.50 ± 4.42

Control 1: rats sacrificed 4 days after induction of acetic acid ulcers; control 2: self-healing in acetic ulcerated rats; control 3: group of rats received sucralfate at a dose of 50 mg/kg; values in the table are expressed as mean ± SEM (standard error on mean); ^ab^*p* < 0.01 and ^abc^*p* < 0.001: statistically significant compared to control 1; ^∗∗^*p* < 0.01 and ^∗∗∗^*p* < 0.001: statistically significant compared to control 2.

**Table 4:** Effect of extracts on ethanol-/stress-induced gastric ulcers.

Groups	Parameters			
	Doses (mg/kg)	Ulcerated surface (mm^2^)	% Healing	Mucus production (mg)
Normal	—	—	—	137.01 ± 4.83
Control 1	—	31.15 ± 2.80	—	129.53 ± 3.15
Control 2	—	59.91 ± 2.74	—	144.63 ± 1.43
Control 3	—	58.60 ± 0.16	2.18	145.23 ± 3.95
Control 4 (sucralfate)	50	4.00 ± 0.22^∗∗∗^^,abc^	93.17	166.15 ± 1.73^∗∗^
*Corchorus olitorius*	200	7.80 ± 0.21^∗∗∗^^,abc^	86.68	155.5 ± 1.10
*Corchorus olitorius*	400	12.50 ± 0.31^∗∗∗^^,abc^	78.66	134.0 ± 0.67
*Solanum nigrum*	200	23.50 ± 0.15^∗∗^^,abc^	59.89	141.30 ± 3.95
*Solanum nigrum*	400	1.06 ± 0.02^∗∗∗^^,abc^	98.19	167.1 ± 1.98^∗∗^
*Vigna unguiculata*	200	3.67 ± 0.10^∗∗∗^^,abc^	93.73	146.28 ± 0.46
*Vigna unguiculata*	400	9.90 ± 0.12^∗∗∗^^,abc^	83.10	146.0 ± 1.11
*Triumfetta pentandra*	200	5.99 ± 0.31^∗∗∗^^,abc^	89.77	168.0 ± 0.59^∗∗^
*Triumfetta pentandra*	400	0.78 ± 0.06^∗∗∗^^,abc^	98.66	125.0 ± 0.41
“nkui” spices	200	47.10 ± 0.50^∗^^,ab^	19.79	127 ± 2.53
“nkui” spices	400	5.01 ± 0.63^∗∗∗^^,abc^	91.45	165.3 ± 0.56^∗∗^
“Yellow soup” spices	200	14.40 ± 0.55^∗∗∗^^,abc^	75.42	146.7 ± 2.33
“Yellow soup” spices	400	8.50 ± 0.44^∗∗∗^^,abc^	85.49	127.0 ± 0.15

Controls 1 and 2: 4 days ulcerated rats; control 3: ulcerated self-healing rats; values in the table are expressed as mean ± SEM (standard error of mean); ^ab^*p* < 0.01 and ^abc^*p* < 0.001: statistically significant compared to control 2; ^∗^*p* < 0.05, ^∗∗^*p* < 0.01, and ^∗∗∗^*p* < 0.001: statistically significant compared to control 3.

**Table 5:** Effect of aqueous extracts on some parameters of oxidative stress and NO level in gastric tissue “unhealed ulcer” model.

Treatments	Dose (mg/kg)	MDA (*μ*mol/g tissue)	GSH (*μ*mol/g tissue)	Catalase (mM H_2_O_2_/min/g tissue)	SOD (units/g tissue)	Nitrites (*μ*mol/g tissue)
Normal	—	39.02 ± 2.39	96.21 ± 3.64	16.84 ± 3.68	18.33 ± 1.64	1.22 ± 0.13
Control 1	—	42.43 ± 7.82	32.23 ± 5.78^+++^	24.27 ± 2.20	1.16 ± 0.32^+++^	0.09 ± 0.01
Control 2	—	44.18 ± 6.37	73.93 ± 1.85^abc,++^	69.66 ± 0.16^abc,+++^	1.82 ± 0.54	0.69 ± 0.22
Control 3 (sucralfate)	50	46.18 ± 0.24	34.80 ± 4.62^∗∗∗^^,+++^	94.32 ± 6.08^abc,^^∗∗∗^^,+++^	10.24 ± 2.93^abc,^^∗∗∗^^,+++^	2.01 ± 1.03^a^
*C. olitorius*	200	22.48 ± 4.40^a,^^∗∗^	35.32 ± 0.13^∗∗∗^^,+++^	10.71 ± 0.7^a,^^∗∗∗^	0.64 ± 0.05^+++^	2.22 ± 0.44^ab^
*C. olitorius*	400	35.56 ± 6.99	74.39 ± 2.01^++^	72.61 ± 0.75^abc,+++^	6.33 ± 0.09^∗∗∗^^,+++^	2.08 ± 0.02
*Solanum nigrum*	200	54.43 ± 0.22	49.31 ± 0.01^ab,^^∗∗∗^^,+++^	28.78 ± 1.84^∗∗∗^	1.15 ± 0.06^+++^	0.73 ± 0.07
*S. nigrum*	400	32.18 ± 0.05	60.52 ± 0.19^+++^	46.71 ± 1.89^abc,^^∗∗∗^^,++^	0.64 ± 0.06^∗∗∗^^,+++^	0.69 ± 0.15
*Vigna unguiculata*	200	50.97 ± 0.33	16.52 ± 0.21^ab,^^∗∗∗^^,+++^	14.16 ± 0.70^∗∗∗^	36.37 ± 0.08^abc,^^∗∗∗^^,+++^	0.88 ± 0.03
*Vigna unguiculata*	400	36.47 ± 0.14	90.39 ± 0.10	28.23 ± 1.14^∗∗∗^	0.76 ± 0.07^∗∗∗^^,+++^	1.73 ± 0.08
*T. pentandra*	200	39.12 ± 0.52	17.42 ± 0.63^a,^^∗∗∗^^,+++^	16.86 ± 3.07^∗∗∗^	1.43 ± 0.15^+++^	1.16 ± 0.05
*T. pentandra*	400	29.08 ± 5.93	180.1 ± 8.31^∗∗∗^^,+++^	22.55 ± 2.46^∗∗∗^	0.97 ± 0.27^∗∗∗^^,+++^	0.82 ± 0.01
“nkui” spices	200	78.34 ± 0.04^abc,^^∗∗∗^^,+++^	141.5 ± 0.21^abc,^^∗∗∗^^,+++^	40.02 ± 3.16^ab,^^∗∗∗^^,++^	0.75 ± 0.06^+++^	2.01 ± 0.70^a^
“nkui” spices	400	72.16 ± 0.35^abc,^^∗∗∗^^,+++^	131.3 ± 0.07^∗∗^^,+++^	32.82 ± 3.17^a,^^∗∗∗^^,++^	0.87 ± 0.04^∗∗∗^^,+++^	0.04 ± 0.02
“Yellow soup” spices	200	37.61 ± 0.70	10.19 ± 0.03^abc,^^∗∗∗^^,+++^	8.76 ± 0.74^ab,^^∗∗∗^	2.64 ± 0.70^+++^	0.25 ± 0.10
“Yellow soup” spices	400	14.55 ± 0.09^ab,^^∗∗∗^^,+^	50.26 ± 0.07^∗∗^	14.99 ± 1.14^∗∗∗^	1.53 ± 0.38^+++^	0.75 ± 0.04

Normal: group that did not undergo ulcer induction; control 1: rats sacrificed on the 4th day after ulcer induction; control 2: ulcerated rats treated with distilled water for 14 days; control 3: group of rats received sucralfate at a dose of 50 mg/kg. The values in the table represent the means ± SEM; ^a^*p* < 0.05, ^ab^*p* < 0.01, and ^abc^*p* < 0.001: statistically significant difference from control 1; ^∗^*p* < 0.05, ^∗∗^*p* < 0.01, and ^∗∗∗^*p* < 0.001: statistically significant difference from control 2; ^+^*p* < 0.05, ^++^*p* < 0.01, and ^+++^*p* < 0.001: statistically significant difference from normal.

**Table 6:** Effects of aqueous extracts on oxidative stress parameters in gastric tissue ethanol/stress model.

Treatment	Dose (mg/kg)	MDA (*μ*mol/g tissue)	GSH (*μ*mol/g tissue)	Catalase (mM H_2_O_2_/min/g tissue)	SOD (unit/g tissue)	Nitrites (*μ*mol/g tissue)
Normal	—	39.02 ± 2.39	142.3 ± 10.57	138.0 ± 2.37	0.86 ± 0.19	0.15 ± 0.01
Control 1	—	32.43 ± 0.82	12.23 ± 1.78^+++^	18.27 ± .1.30	0.86 ± 0.20^+++^	0.07 ± 0.01
Control 2	—	21.32 ± 0.99	152.5 ± 7.89	156.9 ± 6.60	1.62 ± 0.09	0.76 ± 0.01^+^
Control3	—	8.88 ± 2.23^ab,+++^	99.46 ± 3.16^a,+^	154.0 ± 2.37	0.54 ± 0.09	0.70 ± 0.08^+^
Control 4	50	19.08 ± 0.06^∗^^,ab,+++^	77.92 ± 2.30^++^	144.0 ± 1.96	0.77 ± 0.15	0.48 ± 0.04
*Corchorus olitorius*	200	13.44 ± 1.76^++^	76.89 ± 3.00^ab,++^	151.9 ± 2.792	0.69 ± 0.13	0.96 ± 0.19^++^
*Corchorus olitorius*	400	10.37 ± 1.06^abc,+++^	97.70 ± 2.30^a,++^	156.7 ± 6.60	1.40 ± 0.06	0.83 ± 0.18
*Solanum nigrum*	200	11.52 ± 1.64^a^	101.4 ± 0.58^a^	145.7 ± 3.315	0.52 ± 0.08	0.53 ± 0.06
*Solanum nigrum*	400	11.65 ± 0.15^abc,++^	100.3 ± 5.58^a,+^	150.5 ± 2.11	28.26 ± 12.98^ab,^^∗∗∗^^,+^	0.70 ± 0.15
*Vigna unguiculata*	200	63.38 ± 0.97^abc,^^∗∗∗^^,++^	94.02 ± 1.62^a,+^	160.5 ± 0.78	2.91 ± 0.65^a,^^∗∗^^,+^	1.15 ± 0.11^∗^^,++^
*Vigna unguiculata*	400	7.09 ± 0.70^abc,+++^	84.75 ± 1.41^ab,+^	144.9 ± 6.43	0.23 ± 0.04	0.45 ± 0.11
*T. pentandra*	200	9.53 ± 1.58^ab,+++^	106.9 ± 0.11^a,++^	173.6 ± 3.11^+^	2.28 ± 0.17^∗∗^^+^	1.63 ± 0.01^abc,^^∗∗^^,++^
*T. pentandra*	400	25.43 ± 1.84^∗∗∗^	114.8 ± 3.41^+^	231.4 ± 6.32^abc,^^∗∗∗^	1.68 ± 0.11	2.90 ± 0.89^ab,^^∗∗^^,+++^
“Nkui” spices	200	54.27 ± 1.26^abc,^^∗∗∗^^,++^	137.8 ± 9.74^∗∗∗^	165.8 ± 11.4^+^	0.24 ± 0.18^a^	0.51 ± 0.18
“Nkui” spices	400	35.68 ± 1.70^abc,^^∗∗∗^^,+^	69.61 ± 3.06^ab,^^∗∗^^,+++^	189.2 ± 9.16^ab,^^∗∗^^,+^	1.13 ± 0.03	2.47 ± 0.70^∗∗^^,+++^
“Yellow soup” spices	200	23.31 ± 4.03^∗∗∗^	105.9 ± 2.44^a,+^	167.1 ± 4.62^+^	1.05 ± 0.32	1.14 ± 0.11^∗^^,++^
“Yellow soup” spices	400	11.71 ± 0.70^abc,+^	109.9 ± 3.11^a,+^	189.6 ± 2.85^ab,^^∗∗∗^^,+^	3.27 ± 0.31	0.98 ± 0.17^++^

Normal: group that did not undergo ulcer induction; control 1: rats sacrificed on the 4th day after ulcer induction; control 2: rats sacrificed on the 4th day after ulcer induction; control 3: ulcerated rats treated with distilled water for 10 days; control 4: group of rats treated with sucralfate at a dose of 50 mg/kg. The values in the table represent the means ± SEM; ^a^*p* < 0.05, ^ab^*p* < 0.01, and ^abc^*p* < 0.001: statistically significant difference from control 2; ^∗^*p* < 0.05, ^∗∗^*p* < 0.01, and ^∗∗∗^*p* < 0.001: statistically significant difference from control 3; ^+^*p* < 0.05, ^++^*p* < 0.01, and ^+++^*p* < 0.001: statistically significant difference from normal.

## Data Availability

All additional necessary data can be obtained from the corresponding author upon request.
